# A Low-Cost Method Shows Potentially Toxic Element Levels in Dust Correlated with Elevated Blood Levels of These Chemicals in Children Exposed to an Informal Home-Based Production Environment

**DOI:** 10.3390/ijerph192316236

**Published:** 2022-12-04

**Authors:** Fairah Barrozo, Gilmar Alves de Almeida, Maciel Santos Luz, Kelly Polido Kaneshiro Olympio

**Affiliations:** 1 Department of Environmental Health, School of Public Health, University of São Paulo, Sao Paulo 01246-904, Brazil; 2 Advanced Materials, Laboratory of Metallurgical Processes, Institute for Technological Research of the State of São Paulo, Sao Paulo 05508-901, Brazil

**Keywords:** blood, metals, child, environmental exposure, occupational exposure, dust, child health, worker health

## Abstract

Dust is recognized as a route of exposure to environmental pollutants. The city of Limeira, Sao Paulo state, Brazil, is a production center for jewelry and fashion jewelry, where part of this jewelry production is home-based, informal, and outsourced. The aim of this study was to evaluate exposure to Potentially Toxic Elements (PTE: Cr, Sn, Mn, Sb, Ni, Cu, Zn, Cd, Pb, and As) in dust among children from households of informal workers using electrostatic dust cloths (EDC). Dust samples were collected in 21 exposed and 23 control families using EDC from surfaces where dust deposits had accumulated for approximately 14 days. In exposed families, dust samples were also collected from welders’ workstations. PTE concentrations were then determined using inductively coupled mass spectrometry (ICP-MS). The results raised concerns in relation to Cr, As, and Cd exposure among children within the informal home-based production environment. Blood PTE concentrations in children showed a moderate correlation with levels of Cr (Rho 0.40), Zn (Rho −0.43), and As (Rho 0.40), and a strong correlation with Cd (Rho 0.80) (*p* < 0.05), detected in dust. In conclusion, analyzing dust collected using EDC proved a potentially low-cost tool for determining PTE in dust. In addition, the results confirmed that informal home-based work poses a risk for children residing in these households. Public policies are needed to assist these families and promote better conditions of occupational health and safety for the whole family.

## 1. Introduction

Cottage industries are a subgroup of informal work characterized by artisan and craft production, usually with family participants and carried out within homes or backyards [1] as opposed to companies or purpose-built facilities [2]. This type of occupational activity is often outsourced and associated with the use of hazardous substances, such as lead, arsenic, and cadmium [3], with examples including cottage industries, subsistence fishing, artisanal pot making, battery dismantling, artisanal gold mining [2], and jewelry manufacturing.

A study of homes in Zuni Pueblo, New Mexico, a production center for this cottage industry, showed that residual metals from the jewelry-making process were a potential risk for chronic, low-level exposure to metals, such as silver, copper, lead, and cadmium [4]. However, it is difficult to convince workers who develop occupational diseases of the link between their exposure to hazardous materials and resultant illness. Furthermore, all members of the worker’s family can be at risk, including young children and infants residing in the same environment [1]. Young children can be at particular risk because they spend more time at home, especially those engaging in hand-to-mouth behavior [3] and in the habit of eating non-nutritive substances (pica) [3,5].

A previous study investigated exposure to metals among people making cookware in informal foundries in South Africa [6]. The authors found a statistically significant difference in blood lead levels (BLL) between the group of artisanal pot makers and the non-exposed group on quantile regression (*p* = 0.0003). Moreover, the analysis of pot makers’ handwipes pre- and post-work revealed variable exposure to Al, As, Ba, Bi, Cd, Ce, Co, Cr, Cu, Fe, Li, Mn, Mo, Ni, Pb, Sb, Sn, U, V, Zn, and Zr. Another study compared a small group of children with neurological complaints, such as convulsions and drowsiness, to a control group in Mumbai, a city which has a booming artificial jewelry cottage industry [7]. Mean BLL was 42.6 ± 22.5 μg/dL (range 16.6–85.4) in the exposed group versus 8.7 ± 1.2 μg/dL (range 7.0–10.2) in the control group, a statistically significant difference (*p* < 0.001) [7]. In addition, 80.0% of the exposed group had a history of lead smelting activities within the home to produce artificial jewelry, compared with only 35.7% of controls [7].

In Bangladesh, there are lead acid battery manufacturing industries, predominantly cottage industries with small, poorly ventilated environments [8]. A study involving these workers found high blood lead concentrations averaging 65.25 μg/dL, where 84% of the workers had blood Pb concentrations > 40 μg/dL [8]. Moreover, in Indonesia, 69.5% of the children living near a lead acid battery recycling site (deactivated but still working illegally) had high blood lead levels > 10 μg/dL [9]. Also, dust and blood samples of pottery artisans, in Brazil, showed an excessive exposure to Pb during the pottery-glazing process, which 2.3% of the artisans had blood lead levels above 40 μg/dL [10]. The city of Limeira, Brazil, is a large center of jewelry and fashion jewelry production [11] with a unique home-based outsourced informal production process. The exposure scenario is complex, with multiple sources of Potentially Toxic Elements (PTE), such as chrome (Cr), manganese (Mn), nickel (Ni), copper (Cu), zinc (Zn), arsenic (As), cadmium (Cd), antimony (Sn), and lead (Pb). Workers, and especially their children, are exposed to occupational exposure levels [12,13]. Moreover, most workers engaged in soldering and similar activities do not use personal protective equipment (PPE) [12], further increasing exposure levels.

Children breathe air faster as a function of their lower body weight, and their skin more readily absorbs some harmful substances [14]. Children’s immune systems are also more vulnerable, as their organs have not fully matured, especially during the development of the central nervous system between the age of 6 months and 3 years [14,15]. Dust intake by infants and young children is proportionally higher than for other age groups [16]. Therefore, informal, outsourced, home-based jewelry production raises concern because of the potential presence of dust metals from welding processes. Thus, the aim of this study was to evaluate children’s exposure to metals in house dust by determining PTE levels in household dust, through a low-cost method, and correlating them with blood PTE concentrations. Furthermore, the exposure scenario was evaluated, yielding recommendations of actions to protect the children’s health.

## 2. Materials and Methods

### 2.1. Study Population

The present investigation is part of a larger research study, “The ‘omics’ era applied to society: the impact of formal and informal labor on the exposome of workers with an emphasis on metabolomics, transcriptomics and lipidomics” by the Human Exposome Research Group of the School of Public Health, University of São Paulo, which also involved collection of blood, urine, and whole saliva samples from informal workers of Limeira, and of blood and urine from the children who are members of the workers’ families.

**Figure 1:**
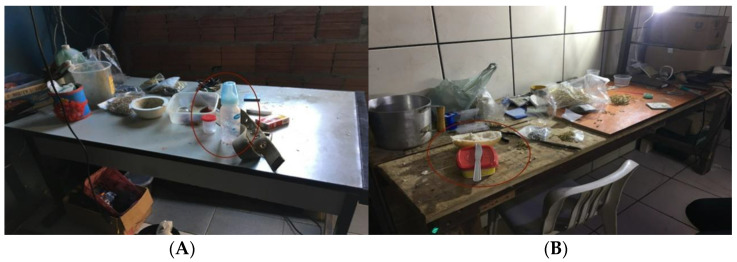
Welder’s workstations with baby feeding bottle (**A**) and food items (**B**). Limeira, 2019.

All of the families participating in this study were volunteers, comprising 21 exposed and 23 control families. The exposed group was made up of families who performed jewelry soldering in the domestic environment. In most cases, the same environment was used for both feeding and work activities, as shown in Figure 1 parts A and B. The families were selected with the assistance of the Health Secretariat of Limeira city. The control group consisted of families with no occupational chemical exposure, selected for invitation by counting the fourth household clockwise facing the street from the exposed households. In the event of refusal to accept the invitation, the neighboring house was invited [12].

### 2.2. Dust Sample Collection

**Figure 2:**
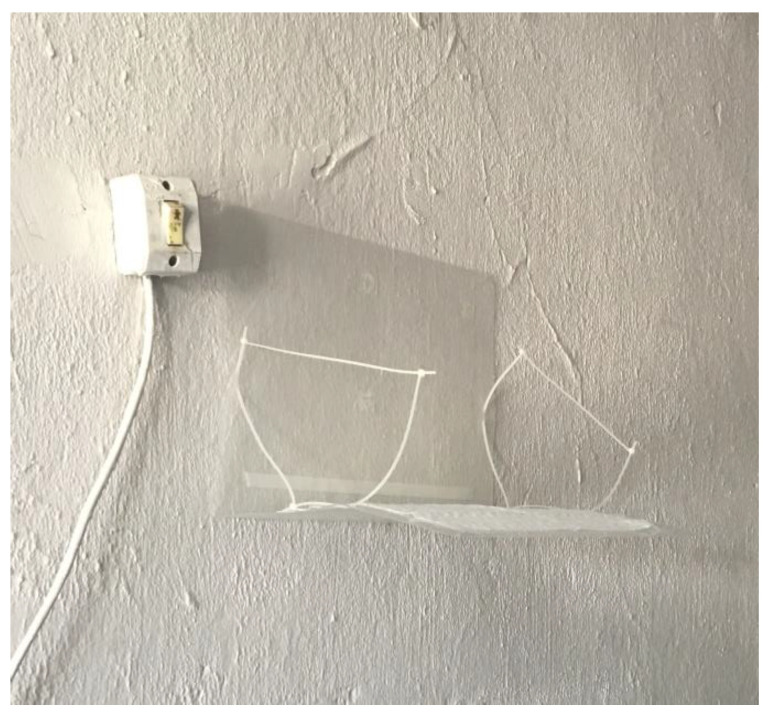
EDC. Limeira, 2019.

Dust samples were collected with EDC (Procter and Gamble^®^) cloths. One EDC was placed inside an open pre-decontaminated plastic folder and affixed to the wall at a height of 1.5 m for 14 to 17 days before the collection of the biological samples (Figure 2). The folder was subsequently closed and transported to the laboratory at ambient temperature [17]. All of the samples were collected in duplicate, labeled for each household, and opened only at the time of analysis to avoid external contamination.

This method was adapted to simulate dust accumulation on furniture because prior visits showed that most families did not have furniture from which dust could be collected. In the workers’ homes, the EDC was placed near the workstations, whereas in the control group, the EDC was placed in the room where the children spent most of their time (bedroom, living room, or kitchen), as informed by the children’s parents or legal guardians.

In addition, surface dust samples were collected directly from welders’ workstations. One EDC was used to clean part of the worktop, stored, and then transported in plastic vessels to the laboratory. All the areas cleaned were measured and recorded.

All of the materials used for sample collection and transport, such as the folders, were previously cleaned using nitric acid overnight and tested for PTE using XRF analyses.

### 2.3. Dust Sample Preparation and Chemical Analysis

All the EDCs were analyzed at the Institute for Technological Research of the State of São Paulo (IPT). For the chemical analysis, the tubes were decontaminated with the addition of 10 mL of 32.5% *m*/*v* nitric acid and heated to a high temperature in a microwave digester. After cooling, the tubes were rinsed with ultrapure water.

The sample preparation involved acid extraction with a microwave oven (Ethos UP model, Milestone). Each EDC sample was cut into four parts with stainless steel scissors previously cleaned with ethyl alcohol. Each part was then placed into a polytetrafluoroethylene tube with plastic forceps and 12 mL of 65% *m*/*v* nitric acid, 2 mL of 35% *m*/*v* hydrogen peroxide, and 0.4 mL of 48% *m*/*v* hydrofluoric acid were then added [18]. The tubes were sealed and placed in the microwave oven. The final solutions were transferred to conical tubes and bulked up with deionized water, and then analyzed by inductively coupled plasma mass spectrometry (ICP-MS) [19]. High purity deionized water was obtained using a Milli-Q water purification system (Millipore, Bedford, MA, USA) and used for the preparation of samples and solutions. The standard material NIST 1648A for Particulate Urban Material [20] was used as certified reference material.

The limits of detection (LOD) and quantification (LOQ), respectively, in μg/cloth, were: Cr (0.1667/0.5154), Mn (0.0049/0.0076), Ni (0.2954/0.3197), Cu (0.0050/0.0085), Zn (0.1124/0.1354), As (0.0032/0.0064), Cd (0.0004/0.0012), Sn (0.0035/0.0084), and Pb (0.0006/0.0011).

### 2.4. Exposure Questionnaires

As part of the exposure assessment, questionnaires were applied to the families regarding the chemicals used, exposure time, work shifts, working environment, ventilation conditions, use of personal protective equipment, and the presence and exposure time of children in the dwellings [21]. Additionally, a work diary was filled out by the workers engaged in soldering to quantify the hours worked during the dust sample collection periods (14 to 17 days), during which the EDC was placed in the homes. To this end, workers reported the time dedicated to soldering work and the place where they worked during the study period.

### 2.5. XRF Measurements

PTEs were determined in the solder powders and wires used for the jewelry and fashion jewelry production at the 17 exposed families’ homes using a Thermo Fisher TM portable X-Ray Fluorescence Analyzer (NitonTM XL2^®^). Not all of the exposed families had solder powders and wires in the residence during the sample collection period.

All the measurements were performed in situ at workers’ homes during the collection of dust samples in October and November 2019.

The analyzer was placed close to the material for 30 s with the trigger pulled [22] and readings were stored in the device until being downloaded to its program software, Standard Thermo Scientific™ Niton Data Transfer (NDT™). Additionally, for quality control, the device was calibrated, every time it was turned on, by standardization and measurement of a specific stainless-steel alloy (alloy 316) [13] and was previously calibrated by the manufacturer before commencement of the study.

### 2.6. Secondary Data Variables

As part of the larger research study by our group, the cited data on blood PTE concentrations of the children were obtained previously [23]. This dataset included concentrations from 29 children, comprising 14 exposed individuals (9 families) and 15 control individuals (14 families) aged 1–11 years. The blood samples were collected at the same time [23] as the dust samples at each house, between October and November 2019.

The blood collection methodology has been described elsewhere [23]. Briefly, the collections were performed by a trained nurse who collected six milliliters of whole blood in heparinized tubes free of trace elements (Vacutainer^®^). The biological samples were then stored at 80 °C before being transferred to the laboratory of the IPT, where determination of PTE was carried out using ICP-MS, in the same manner as for the dust samples.

### 2.7. Data Processing and Statistical Treatment

All statistical analyses were performed using the R and RStudio statistical package (version 1.3.1093). The concentrations of metals analyzed in the dust were expressed using descriptive statistics, including minimum and maximum, standard deviation, geometric mean (GM), and the 95th percentiles of each element. Participants were stratified by exposure group (exposed or control). In addition to comparison tests, Student’s *t*-test and the Mann–Whitney test were performed, according to the distribution of the variables. Results were considered statistically significant for *p*-values < 0.05. Nonparametric Spearman correlations were computed to assess univariate correlations of PTE levels in house dust with concentrations in children’s blood, number of workers and work time, and also for different dust sample collection methods.

Elemental concentrations in blood and dust samples below the limit of detection (LOD) were assigned a value of LOD/2 [24].

## 3. Results

The participants (n = 44) comprised 21 exposed and 23 control families. One of the exposed family used two rooms for working and three workstations. Correspondingly, two dust samples were collected from the rooms for approximately 14 days after installation of EDC (14 days before blood sample collection) and three dust samples were collected from the welders’ workstations. Most of the welders were women (n = 41). Regarding the exposed group, the welders reported using chemical products during their work process, such as acid and soldering powder (23.8%), acid and soldering wire (23.8%), and soldering powder alone (47.6%). Furthermore, only 19.0% of the workers who engaged in soldering stated that they used PPE during the work, such as safety glasses, dust masks or respirators, face shields, gloves, and protective clothing. Overall, 42.9% of the welders stated that they used ventilation systems, while 80.9% worked with doors and windows open or in the open air. A total of 80.9% of welders stated that they worked in living rooms, bedrooms, laundry rooms, and backyards, as shown in Table 1. In 42.9% of households, welding activities were shared by spouses, siblings, and friends.

PTE concentrations in the house dust samples collected over approximately 14 days were significantly higher (*p* < 0.05) in the exposed group for all PTE included in this study, but levels of the elements detected in these accumulated dust samples (worktop cleaning) from homes in the exposed group were not correlated with concentrations found in welders’ workstation dust, except for Sn (*p* < 0.05) (Table 2).

The PTE concentrations in Table 2 were calculated based on the area cleansed for the samples collected from welders’ workstations and on the exposed EDC area for the accumulated dust samples.

The levels of Pb detected did not exceed the LOD in any of the dust samples collected for approximately 14 days. Additionally, PTEs in solder powders and wires were determined using a field-portable X-ray fluorescence analyzer, except for As. The following values for Cd (geometric mean (GM): 53,927.21 ppm; standard deviation (SD): 32,806.44 ppm; range: 5209.72–165,296.14 ppm), Cr (GM: 4115.78 ppm; SD: 2810.68 ppm; range: 465.31–14,550.51 ppm), Mn (GM: 1161.33 ppm; SD: 460.50 ppm; range: 1050.71–1471.06 ppm) and Ni (GM: 524.22 ppm; SD: 291.83 ppm; range: 102.03–1015.37 ppm) were found for solder powders only, while a value of Pb (GM: 249,973.13 ppm; SD: 165,157.41 ppm; range: 5840.90–522,785.34 ppm) was determined for solder wires only (Table 3), consistent with the results found in the dust exposure samples. Additionally, Cu and Zn were higher in solder powders and Sn was higher in solder wires.

Spearman correlations between PTE concentrations in dust samples and the number of welders working in the same room and work time were not statistically significant (*p* > 0.05).

Moreover, Spearman correlations between PTE concentrations and the physical infrastructure of the workspaces (area, doors and windows areas, and ceiling height) were heterogeneous (Table 4). The correlation of PTE determined in dust deposits (cleaning worktop) (exposed group) with the area was statistically significant for Zn (Spearman correlation coefficient: Rho = 0.57) and Sn (Rho = 0.54), with door area for Mn (Rho = 0.71) and Sn (Rho = 0.45), and with ceiling height for Zn (Rho = 0.47), *p* < 0.05. Regarding the control group, correlations of PTE determined in dust deposits were statistically significant (*p* < 0.05) only with ceiling height for Cr (Rho = 0.48), Mn (Rho = 0.44), As (Rho = 0.65) and Sn (Rho = 0.46). Although PTE concentrations in welders’ workstation dust proved statistically significant (*p* < 0.05) with area for Ni (Rho = 0.45) and Zn (Rho = 0.44) and with door area for Zn (Rho = 0.42), the correlation between window area and PTE dust deposits was not statistically significant (*p* > 0.05).

### Socio-Demographic Data and Blood PTE Levels of Children

The subgroup of children, members of the families assessed, comprised 14 exposed and 15 control children aged 1–11 years. The mean age of the exposed group was 6 years (range: 1–11 years) comprising 4 females and 10 males, whereas the mean age of the control group was 6 years (range: 2–11 years) comprising 8 females and 7 males, showing the homogeneity of participants for the exposure groups. The mean time staying at home during the week for the children was approximately 7 h in both groups, ranging from 3–24 h for the exposed group and 10–24 h for the control group.

The blood samples of the exposed group had a mean and standard deviation (SD) of 1.62 µg/L (0.42), 9.9 µg/L (3.63), 3.35 µg/L (1.03), 1147.31 µg/L (278.13), 3698.25 µg/L (1155.84), 0.40 µg/L (0.19), 0.11 µg/L (0.11), 6.42 µg/L (20.99), and 3.31 µg/dL (2.29) for Cr, Mn, Ni, Co, Zn, As, Cd, Sn, and Pb, respectively. For the control group, the mean (SD) levels in blood were 1.49 µg/L (0.39), 10.43 (3.41), 3.23 µg/L (0.68), 1204.49 µg/L (205.56), 4227.96 µg/L (696.02), 0.34 µg/L (0.22), 0.01 µg/L (0.01), 0.80 µg/L (0.23), and 1.31 µg/dL (0.53) for Cr, Mn, Ni, Co, Zn, As, Cd, Sn, and Pb, respectively. The results showed a statistical difference for Cd and Pb (*p* < 0.05) between the control and exposed groups, as shown in Table 5.

Finally, the correlations between concentrations of PTEs in the blood samples and levels in dust deposits collected for approximately 14 days were moderate for Cr, Zn, and As, and strong for Cd. No statistically significant correlations were found between PTE levels in the blood and in dust collected from welders’ workstations (*p* > 0.05) (Table 6).

## 4. Discussion

To the best of our knowledge, there are few studies [25] investigating the use of EDC in the assessment of PTE exposure, where most studies use EDC to investigate microbiological exposure. However, some studies have used the cloth method to analyze exposure to metals by surface cleaning [26,27]. During the soldering process, workers are often exposed to pollutants, ash and dust, fumes, and hazardous chemicals [28]. The present study found moderate (Cr Rho = **0.40**, As Rho = **0.40**) and strong (Cd Rho = **0.80**) positive correlations between levels of these PTEs in children’s blood and in dust deposits collected for approximately 14 days (*p*-value < 0.05), confirming that PTE concentrations in blood can be influenced by dust concentrations. Oral contact is the most common route of exposure, but inhalation is also an important route [12]. The dust collected from welders’ workstations proved to be a less relevant exposure route (*p* > 0.05) for all PTE except Sn. This finding can be explained by the complexity of the informal work scenario, with no fixed workstations or number of workers and the presence of unregistered cleaning routines or accidents related to handling soldering powder, such as dropping on floors and furniture, in the present study. Despite cleaning routines, the hazard of high PTE concentrations in dust from the floor or the air which can be inhaled or ingested by children remains.

In a previous study, 19 ash and dust samples were collected into glass beakers from jewelry workshops in Bangladesh and high concentrations of the compounds Cd, Cr, Pb, and As were found [28]. Other studies also showed that high metal concentrations in inexpensive jewelry [29,30] were a health concern. High metal concentrations can also pose a health risk after the production process during use of the items by children. High Pb concentrations in low-cost jewelry from Cambodia were detected using X-ray fluorescence (XRF) measurements [31]. These health risks can also be found in developed countries, where high Cd in inexpensive jewelry using XRF measurements was found [29]. Even in jewelry with high surface Cd levels, bioavailability to the wearer during dermal contact was low, although the same does not apply to manufacturing of the jewelry, since solder powder has a high Cd concentration [32].

The results of the present study showed statistically significant differences (*p* < 0.05) between exposed and control groups for blood levels of the metals Cd (mean: Control 0.001 µg/L; Exposed 0.011 µg/L) and Pb (mean: Control 1.31 µg/dL; Exposed 3.31 µg/dL). However, on the analysis of blood and dust PTE levels, Spearman correlations revealed statistically significant differences (*p* < 0.05) for Cr, Zn, As, and Cd in dust samples collected for approximately 14 days. A study analyzed associations for isotopic ratios in comparisons between lead concentrations in blood samples from 30 children and environmental samples (floor dust, soil, drinking water and paint) in Australia [33]. The authors concluded that floor dust collected using EDC showed the most significant correlation, as evidenced by regression analyses.

Surface dust samples were collected from the homes of Native American jewelry makers and from homes in which jewelry was not made in New Mexico [4]. The dust samples were collected by cleaning wall and floor areas near the workplace and the dining rooms of control participants, using Whatman^®^ filter paper. The concentrations of Ag, Cu, Ni, Mg, and Sb were significantly higher in exposed homes than in control homes (*p* ≤ 0.02). Moreover, Ag, Cu, Sn, B, Ni, Zn, Pb, and Cd concentrations were significantly higher in samples collected in work areas when compared with living areas in exposed homes (*p* = 0.02, paired *t*-test). These findings are corroborated by the results of the present study, revealing significantly higher concentrations in samples of accumulated dust in the exposed group for all PTEs assessed, except Pb (*p* < 0.05).

The Pb concentrations detected in accumulated dust were below the LOD. Although Pb was found only in soldering wires and not soldering powders, all of the materials (soldering powders and wires, jewelry, and acids) are employed informally and have no clear provenance and/or instructions on use and safety. Blood lead levels (BLL) of the children in the exposed group reached 7.5 μg/dL, and the US Centers for Disease Control and Prevention (CDC), based on results from the National Health and Nutrition Examination Survey (NHANES), has determined a Blood Lead Reference Value (BLRV) of 3.5 μg/dL [34]. The BLRV of the CDC serves as a guide to determine medical or environmental actions and prioritize communities for actions preventing exposure. No safe BLL in children exists, where even low levels can cause harm [35]. Children are especially sensitive to Pb damage, principally because their central nervous system is still developing and more vulnerable to toxic agents [5]. In Latin America, there is a dearth of legal instruments on exposure to lead [36]. In a study of 29 children (15 cases and 14 controls) from Mumbai exposed to a jewelry cottage industry, the authors described cases with neurological symptoms of seizures (n = 12) and drowsiness (n = 3), and mean blood lead level of cases was 42.6 ± 22.5 μg/dL (range 16.6–85.4 μg/dL) proving significantly higher in these exposed children than in controls (mean BLL 8.7 ± 1.2 μg/dL, range 7.0–10.2 μg/dL) (*p* < 0.001) [7].

A previous study of the same population of welders analyzed PTE concentrations in air of the breathing zone of the workers and in the blood of the workers and their relatives within the same household, raising concerns about the concentrations of Ni and Cd [12]. Moreover, PTEs are often intentionally added to jewelry items because they are good coating agents, can lower manufacturing costs, improve workability, produce shiny surfaces, and mimic famous jewelry items [30]. Blood PTE concentrations in adults from the same population were measured, showing higher blood levels in the exposure group for As (0.44 μg L^−1^), Cd (0.21 μg L^−1^) and Pb (1.88 μg L^−1^) compared to the control group (0.35 μg L^−1^, 0.01 μg L^−1^ and 1.04 μg L^−1^ for As, Cd, and Pb, respectively) [23].

Regarding physical infrastructure, correlations between physical infrastructure and dust PTE concentrations in the present investigation were heterogeneous. Another study measured the accumulation rate of ash and dust in jewelry workshops in Bangladesh by weighing the passive samples, the calculated accumulation rate of ash showed no strong correlation with workshop size or number of jewelry workers [28]. However, a slight difference was observed between the different types of manufacturing units, where smelting, polishing, cutting, and enameling units produced more ash and dust than soldering activities. It is important to note that a large number of households did not have appropriate workspaces, with rooms divided using cloth and cardboard, potentially impacting air circulation and results.

This study has some limitations. First, dust and children’s blood sampled only once during the year, where the relationships observed between study variables and PTE concentrations may vary by season and work demand. Second, the number of participants was small, preventing different interactions from being further explored. The primary reason for parents or legal guardians refusing to participate was not wanting their child to have a venous blood draw. Third, only one exposure pathway was analyzed, namely, dust, but other pathways such as water, soil from the unpaved yard, and paint from the walls should be further analyzed. However, accessing home-based welders is difficult because most live in areas of high social vulnerability, change work constantly, and principally, the production of informal jewelry is not regulated by law and, therefore, most workers do not want to be exposed to authorities. Last, some studies have used different kinds of cloths to analyze metal exposure by cleaning surfaces [26,37], but Swiffer cloths were used in the present study as they are both accessible and low-cost and have proven effective for the proposed application.

## 5. Conclusions

The study results highlighted concerns regarding children’s exposure to Cr, As, and Cd within the informal home-based environment. It is important to foster actions promoting safety, including preventing the involvement of children in this type of labor and restricting their access to workspaces and materials. In addition, EDC represents a potentially low-cost tool for evaluating PTE exposure, particularly in highly vulnerable areas and in studies with limited funding.

## Figures and Tables

**Table 1:** Distribution of demographic characteristics and working conditions of study population by group. Limeira, Brazil, 2019.

Characteristics	N	%
Total number of participating families	44	100.0
Exposed	21	47.7
Control	23	52.3
Total number of participating children	29	100.0
Exposed	14	48.3
Control	15	51.7
Exposed group	21	
Chemicals used		
Acid and soldering powder	5	23.8
Acid and soldering wire	5	23.8
Soldering powder alone	10	47.6
None	1	4.8
Use of personal protective equipment		
Yes	4	19.0
No	17	80.9
Use of ventilation system		
Yes	9	42.9
No	12	57.1
Place of work		
Living room	3	14.3
Kitchen	1	4.8
Bedroom	2	9.5
Laundry	1	4.8
Work room	4	19.0
Open area (backyard)	10	47.6
Use of natural ventilation		
All doors and windows left open	17	80.9
Doors open and windows closed	2	9.5
Depends on temperature	1	4.8
Other	1	4.8

**Table 2:** PTE levels (μg/cm^2^) in dust samples by group. Limeira, Brazil, 2019.

	Accumulated Dust ^a^							Welders’ Workstation Dust ^b^		
	Exposed		Control		Total			Exposed		
	N = 21		N = 23		N = 44			N = 21		
	Mean	SD	Mean	SD	Mean	SD	*p*-Value ^c^	Mean	SD	*p*-Value ^d^
Cr	0.0061	0.0130	0.0004	0.0003	0.0032	0.0094	<0.05	0.11	0.53	>0.05
Mn	0.0052	0.0034	0.0014	0.0011	0.0033	0.0031	<0.05	0.01	0.03	>0.05
Ni	0.3979	0.2099	0.0005	0.0003	0.1948	0.2478	<0.05	0.02	0.11	>0.05
Cu	0.0044	0.0034	0.0004	0.0006	0.0024	0.0031	<0.05	0.26	0.73	>0.05
Zn	0.3483	1.3181	0.0002	0.0000	0.1704	0.9274	<0.05	0.46	0.91	>0.05
As	0.0002	0.0001	0.0000	0.0000	0.0001	0.0001	<0.05	0.00	0.00	>0.05
Cd	0.0017	0.0028	0.0000	0.0000	0.0009	0.0021	<0.05	0.03	0.09	>0.05
Sn	0.0529	0.2105	0.0001	0.0002	0.0259	0.1479	<0.05	0.07	0.17	<0.05
Pb	<LOD	<LOD	<LOD	<LOD	<LOD	<LOD	-	0.06	0.13	-

SD—Standard Deviation; <LOD—Below limit of detection. ^a^ Dust collected for approximately 14 days after installation of EDC. ^b^ Dust collected from welders’ workstations. ^c^ Mann–Whitney test in exposed and control groups for dust deposits on EDC after approximately 14 days. ^d^ Spearman correlation between accumulated dust deposits on EDC after approximately 14 days and dust deposits on welders’ workstations.

**Table 3:** PTE levels (ppm) in soldering powders and wires. Limeira, Brazil, 2019.

	Soldering Powders N = 13			Soldering Wires N = 4		
	GM ^a^	SD ^b^	Min–Max	GM ^a^	SD ^b^	Min–Max
Cd	53,927	32,806	5209–165,296	<LOD	<LOD	<LOD
Mn	1161	460	1050–1471	<LOD	<LOD	<LOD
Ni	524	291	102–1015	<LOD	<LOD	<LOD
Cu	314,720	101,971	34,242–497,231	1800	8155	546–23,599
Zn	201,336	68,072	19,732–317,607	873	1159	202–2931
Cr	4115	2810	465–14,550	<LOD	<LOD	<LOD
Sn	1462	1244	635–5743	597,797	157,267	473,193–966,567
Pb	<LOD	<LOD	<LOD	249,973	165,157	5,840–522,785

<LOD—Below limit of detection. ^a^ GM—Geometric mean. ^b^ SD—Standard Deviation.

**Table 4:** *p*-Values of Spearman correlations between PTE levels in dust samples and physical infrastructure by group. Limeira, Brazil, 2019.

	Accumulated Dust ^a^																Welders’ Workstation Dust ^b^							
	Area				Window Area				Door Area				Ceiling Height				Area		Window Area		Door Area		Ceiling Height	
	Exposed		Control		Exposed		Control		Exposed		Control		Exposed		Control		Exposed N = 21							
	N = 21		N = 23		N = 21		N = 23		N = 21		N = 23		N = 21		N = 23									
	Rho	*p*-Value	Rho	*p*-Value	Rho	*p*-Value	Rho	*p*-Value	Rho	*p*-Value	Rho	*p*-Value	Rho	*p*-Value	Rho	*p*-Value	Rho	*p*-Value	Rho	*p*-Value	Rho	*p*-Value	Rho	*p*-Value
Cr	0.10	>0.05	−0.29	>0.05	−0.43	>0.05	0.08	>0.05	0.20	>0.05	−0.26	>0.05	−0.17	>0.05	0.48	**<0.05**	0.20	>0.05	−0.22	>0.05	0.29	>0.05	0.02	>0.05
Mn	0.40	>0.05	−0.34	>0.05	0.04	>0.05	−0.04	>0.05	0.71	**<0.05**	0.01	>0.05	−0.33	>0.05	0.44	**<0.05**	−0.08	>0.05	−0.15	>0.05	−0.10	>0.05	0.08	>0.05
Ni	0.30	>0.05	−0.11	>0.05	−0.33	>0.05	0.12	>0.05	0.03	>0.05	0.29	>0.05	0.12	>0.05	0.00	>0.05	0.45	**<0.05**	−0.04	>0.05	0.27	>0.05	0.29	>0.05
Cu	−0.09	>0.05	−0.08	>0.05	0.12	>0.05	0.08	>0.05	−0.06	>0.05	−0.05	>0.05	−0.37	>0.05	0.33	>0.05	0.13	>0.05	−0.11	>0.05	0.10	>0.05	0.05	>0.05
Zn	0.57	**<0.05**	0.22	>0.05	0.22	>0.05	0.00	>0.05	0.33	>0.05	−0.16	>0.05	0.47	**<0.05**	0.10	>0.05	0.44	**<0.05**	0.07	>0.05	0.42	**<0.05**	0.03	>0.05
As	0.39	>0.05	−0.01	>0.05	−0.45	>0.05	0.18	>0.05	0.38	>0.05	−0.09	>0.05	−0.19	>0.05	0.65	**<0.05**	0.06	>0.05	−0.39	>0.05	−0.10	>0.05	0.24	>0.05
Cd	−0.19	>0.05	−0.15	>0.05	0.05	>0.05	−0.36	>0.05	−0.22	>0.05	0.08	>0.05	−0.36	>0.05	−0.17	>0.05	0.04	>0.05	0.01	>0.05	0.06	>0.05	−0.26	>0.05
Sn	0.54	**<0.05**	−0.31	>0.05	0.14	>0.05	−0.13	>0.05	0.45	**<0.05**	0.05	>0.05	0.30	>0.05	0.46	**<0.05**	0.25	>0.05	−0.29	>0.05	0.32	>0.05	0.21	>0.05
Pb	<LOD	<LOD	<LOD	<LOD	<LOD	<LOD	<LOD	<LOD	<LOD	<LOD	<LOD	<LOD	<LOD	<LOD	<LOD	<LOD	0.30	>0.05	−0.19	>0.05	0.24	>0.05	0.26	>0.05

^a^ Dust collected for approximately 14 days after installation of EDC. ^b^ Dust collected from welders’ workstations. <LOD—Below limit of detection. **<0.05** represents the statistically significant correlations.

**Table 5:** Blood PTE levels determined in children. Limeira, Brazil, 2019.

	Exposed			Control			Total			
	N = 14			N = 15			N = 29			
	Mean	SD	95th Percentile	Mean	SD	95th Percentile	Mean	SD	95th Percentile	*p*-Value *
Cr (µg/L)	1.62	0.42	2.30 (95)	1.49	0.39	2.05 (95)	1.55	0.40	2.27 (95)	>0.05
Mn (µg/L)	9.91	3.63	16.07 (95)	10.43	3.41	16.93 (95)	10.18	3.46	16.64 (95)	>0.05
Ni (µg/L)	3.35	1.03	5.05 (95)	3.23	0.68	4.13 (95)	3.28	0.85	4.81 (95)	>0.05
Cu (µg/L)	1147.31	278.13	1554.40 (95)	1204.49	205.56	1621.93 (95)	1176.90	240.60	1626.90 (95)	>0.05
Zn (µg/L)	3698.25	1155.84	6058.62 (95)	4227.96	696.02	5239.38 (95)	3972.00	966.98	5907.13 (95)	>0.05
As (µg/L)	0.40	0.19	0.78 (95)	0.34	0.22	0.62 (95)	0.37	0.21	0.83 (95)	>0.05
Cd (µg/L)	0.11	0.11	0.30 (95)	0.01	0.01	0.02 (95)	0.06	0.10	0.26 (95)	**<0.05**
Sn (µg/L)	6.42	20.99	28.51 (95)	0.80	0.23	1.08 (95)	3.61	14.85	1.12 (95)	>0.05
Pb (µg/dL)	3.31	2.29	7.14 (97.5)	1.31	0.53	2.17 (97.5)	2.28	1.90	6.66 (97.5)	**<0.05**

* Mann–Whitney tests in exposed and control groups, except Cr, Cu, and Zn, for which Student’s *t*-test was applied. **<0.05** represents the statistically significant differences.

**Table 6:** Spearman correlations of PTE levels in children’s blood (0–11 years) and in dust samples. Limeira, Brazil, 2019.

	Accumulated Dust ^a^		Welders’ Workstation Dust ^b^	
	Total		Exposed	
	N = 29		N = 14	
	Rho	*p*-Value	Rho	*p*-Value
**Cr**	**0.40**	**<0.05**	−0.06	>0.05
Mn	−0.11	>0.05	0.05	>0.05
Ni	−0.04	>0.05	−0.46	>0.05
Cu	−0.22	>0.05	0.37	>0.05
**Zn**	**−0.43**	**<0.05**	−0.03	>0.05
**As**	**0.40**	**<0.05**	−0.05	>0.05
**Cd**	**0.80**	**<0.05**	0.08	>0.05
Sn	0.22	>0.05	−0.18	>0.05
Pb	-	-	−0.43	>0.05

^a^ Dust collected for approximately 14 days after installation of EDC. ^b^ Dust collected from welders’ workstations. Bold represents the statistically significant differences

## Data Availability

Not applicable.
